# Can Neoadjuvant Chemotherapy Cause Postoperative Hydronephrosis After Radical Cystectomy?

**DOI:** 10.7759/cureus.57306

**Published:** 2024-03-31

**Authors:** Sinan Celen, Yusuf Ozlulerden, Aykut Baser, Okan Alkış, Kursat Kucuker, Mesut Berkan Duran

**Affiliations:** 1 Urology, Pamukkale University School of Medicine, Denizli, TUR; 2 Urology, Bandirma Onyedi Eylul University School of Medicine, Balikesir, TUR; 3 Urology, Kutahya Health Science University, Kütahya, TUR

**Keywords:** neoadjuvant chemotherapy(nact), urinary diversion, radical cystectomy, obstructive hydronephrosis, bladder cancer

## Abstract

Objective: This study's objective is to assess the effect of preoperative factors on postoperative hydroureteronephrosis (HUN) after radical cystectomy (RC) in patients with bladder cancer (BC).

Methodology: Patients who underwent RC for BC between January 2019 and November 2022 and had unilateral or bilateral postoperative HUN were retrospectively analyzed. Patients without preoperative HUN but with postoperative HUN constituted the patient group, while patients without both preoperative and postoperative HUN constituted the control group, and they were compared with each other.

Results: Neoadjuvant chemotherapy (NAC) and postoperative metastasis were positively correlated with postoperative HUN (*r* = 0.238, *P* = 0.007, and *r* = 0.203, *P* = 0.021, respectively). Multivariate logistic regression analysis showed that the postoperative HUN was significantly associated with NAC (*P *= 0.048; Exp(*B*) = 6.896, 95% confidence interval [CI] 1.02-46.9) but not associated with the presence of metastasis (*P* = 0.054). Moreover, NAC increased the possibility of undergoing revision surgery (*P* = 0.002; Exp(*B*) = 26.9, 95% CI 3.2-225).

Conclusions: NAC is an independent factor for impaired anastomotic healing, increased postoperative HUN, and the need for revision surgery in patients with BC.

## Introduction

Bladder cancer (BC) is common and fatal worldwide. According to one recent report, the prevalence of BC ranks tenth across the world, with an estimated 573,000 new cases and 213,000 deaths each year [1]. The majority of the disease is transitional epithelial cell carcinoma, which accounts for up to 86.0% of cases [2]. Radical cystectomy (RC) with lymph node dissection is a major treatment option for muscle-invasive BC (MIBC), as well as a therapy option for non-MIBC with very high-risk features [3]. Despite undergoing RC, up to 50% of patients with BC still experience cancer recurrence and death after RC [4].

Hydroureteronephrosis (HUN) is a regular complication of RC with urinary diversion. Hydronephrosis may be linked to worse outcomes for MIBC patients undergoing RC [5]. Preoperative HUN is a prevalent occurrence in MIBC patients, with a frequency of up to 57.9% [6]. Recent studies have shown that a risk factor associated with worse outcomes in renal function is postoperative HUN with ureteral obstruction [7,8]. However, risk factors associated with developing HUN and adverse impacts on anastomotic healing after ureteroileal anastomosis are not well defined. Thus, this study aimed to investigate the factors of postoperative HUN risk after RC.

## Materials and methods

Patients who underwent open RC and extended lymph node dissection with the same technique by the same surgeon at our tertiary referral center between January 2019 and November 2022 were retrospectively reviewed. Operative, pathological, and follow-up clinical data of these patients were recorded. The exclusion criteria were the presence of preoperative HUN or the presence of incomplete data regarding the preoperative-postoperative condition of the patients. Moreover, patients who were not followed up for at least 12 months or were followed up at another center, and those whose HUN was not monitored with computed tomography (CT) or ultrasound (US), were excluded. Furthermore, patients with ureteral obstruction due to metastasis, variant histology, positive surgical margins in the final pathology, and those who underwent RC due to non-MIBC were also excluded from the study. Patients meeting the study criteria were divided into two groups: patients without preoperative HUN but with postoperative HUN constituted the patient group (Group 1), while patients without both preoperative and postoperative HUN constituted the control group (Group 2). HUN was defined as an anteroposterior diameter of the renal pelvis >10 mm by US or CT and the presence of ureteral dilatation (>5 mm) [9].

This retrospective study was approved by the local ethics committee (approval number E. 28284).

Statistical analysis

IBM SPSS Statistics for Windows, Version 22.0 (IBM Corp, Armonk, NY) was used for data analysis. The Kolmogorov-Smirnov test was used to evaluate the normality of the data. Mean, standard deviation, median, minimum, maximum, frequency, number, and percentage were used as descriptive statistics. When comparing the data of two independent groups, either the Student's t-test or the Mann-Whitney U test was used depending on the distribution of the data. A chi-square test was used to compare categorical data between groups, and Fisher's exact test was used in cases where the chi-square test was not appropriate. The relationship between neoadjuvant chemotherapy (NAC), postoperative metastatic disease, and postoperative HUN was evaluated using Spearman correlation analysis. Univariate analyses to identify variables associated with postoperative HUN (yes/no) were investigated using Chi-square, Fisher's exact test, Student's t-test, and Mann-Whitney U test, where appropriate. The possible factors identified with univariate analyses were further entered into the logistic regression analysis to determine independent predictors of postoperative HUN (yes/no) for the multivariate analysis. Hosmer-Lemeshow goodness-of-fit statistics was used to assess model fit. An overall 5% type I error was used for statistical significance.

## Results

Two hundred thirty-five patients underwent RC between January 2019 and November 2022. One hundred and six patients were excluded from the study according to the exclusion criteria, and a total of 129 patients were included in the study. In HUN, follow-ups were performed with CT or US. As a study group, 21 patients who developed dilatation in the postoperative period without dilatation in the preoperative period (Group 1) and 108 patients who did not develop dilatation during the preoperative period and at least 12 months of follow-up were included in the study as the control group (Group 2). Patients’ characteristics, preoperative and postoperative data, and statistical analysis between these groups are presented in Table 1.

**Table 1:** Patient characteristics, preoperative and postoperative data, and statistical analysis according to the presence of postoperative HUN. Group 1 had postoperative HUN but no preoperative HUN. Group 2 had no postoperative HUN and no preoperative HUN. HUN, hydroureteronephrosis

		The presence of postoperative hydronephrosis		P
		Group 2	Group 1	
Age (years)		63.70 ± 9.81	63.19 ± 6.69	0.769
Preoperative creatinine (mg/dL), median (min.-max.)		0.86 (0.50-1.64)	0.90 (0.62-2.71)	0.888
Hospitalization (days), median (min.-max.)		13 (8-30)	13 (8-30)	0.682
Gender, *n* (%)	Male	94 (87.0)	20 (95.2)	0.463
	Female	14 (13.0)	1 (4.8)	
Neoadjuvant chemotherapy, *n* (%)	No	106 (98.1)	18 (85.7)	0.030
	Yes	2 (1.9)	3 (14.3)	
Adjuvant chemotherapy, *n* (%)	No	56 (51.9)	8 (38.1)	0.249
	Yes	52 (48.1)	13 (61.9)	
Adjuvant radiation therapy, *n* (%)	No	92 (85.2)	18 (85.7)	1.000
	Yes	16 (14.8)	3 (14.3)	
Urinary diversion type, *n* (%)	Non-continent	79 (73.1)	13 (61.9)	0.297
	Continent	29 (26.9)	8 (38.1)	
Metastatic disease in postoperative follow-up, *n* (%)	No	83 (76.9)	11 (52.4)	0.021
	Yes	25 (23.1)	10 (47.6)	
Lymph node metastasis, *n* (%)	N0	87 (80.6)	18 (85.7)	0.814
	N1	54 (6.0)	1 (4.8)	
	N2	16 (14.8)	2 (9.5)	
Postoperative T stage, *n* (%)	Ta/T1 + CIS	23 (21.1)	8 (38.1)	0.239
	T2	28 (25.9)	2 (9.5)	
	T3	44 (40.7)	9 (42.9)	
	T4	13 (12.0)	2 (9.5)	

There was no significant difference between the two groups in terms of preoperative serum creatinine levels (*P* = 0.888). Metastatic disease in postoperative follow-up was significantly more common in Group 1 than in the control group (*P *= 0.021). However, no significant difference was found between groups in terms of postoperative tumor (T) stage, lymph node stage (N), and history of adjuvant radiation therapy (*P *= 0.239, 0.814, and 1.000, respectively). NAC and postoperative metastatic disease were positively correlated with postoperative HUN (*r *= 0.238, *P *= 0.007, and *r *= 0.203, *P *= 0.021, respectively). Multivariate logistic regression analysis showed that the postoperative HUN was significantly associated with the history of NAC (*P *= 0.048; Exp(*B*) = 6.896, 95% confidence interval [CI] 1.02-46.9) but not associated with the presence of metastatic disease in postoperative follow-up (*P *= 0.054, Exp(*B*) = 2.672, 95% CI 0.985-7.244). When investigating the relationship between receiving neoadjuvant chemotherapy (NAC) and the necessity of revision surgery, it was found that revision surgery was statistically significantly more frequent in patients who underwent NAC (*n *= 2, 40%) compared to those who did not (*n *= 1, 2.4%) (*P *= 0.012). A positive correlation was found between receiving NAC and the need for revision surgery (*r *= 0.376, *P* <0.001). In the multivariate logistic regression analysis, it was found that receiving NAC increased the possibility of undergoing revision surgery by 26.9 times (*P *= 0.002, Exp(*B*) = 26.9, 95% CI 3.2-225).

## Discussion

Postoperative HUN, which occurs as a complication after RC, may cause renal dysfunction and difficulties in patient management. Moreover, invasive procedures may be necessary in the treatment of this disorder and may create additional physical and psychological difficulties for the patient. To the best of our knowledge, this is the first study to investigate the effect of pre-peri-postoperative parameters on the presence of postoperative HUN in patients with RC. The main finding of the study is that NAC is an independent factor for increased postoperative HUN, and the need for revision surgery because of possible decreased anastomotic wound healing. Moreover, postoperative metastatic disease was significantly correlated with postoperative HUN but lost its statistical significance in multivariable analysis.

Wound healing is a complex process consisting of three stages: inflammation phase, productive phase, and anaplastic phase. Any deficiency at these stages will result in a lack of proper healing of the anastomosis and incomplete structural and functional tissue rehabilitation. Anastomotic healing may be affected by various local or systemic factors such as the age of the patients, comorbidities (such as diabetes mellitus), drugs, nutritional status, and chemoradiation therapy. Moreover, surgical technique, adequate oxygenation of tissues, and infections affect wound healing as local factors [10-12]. Removal of necrotizing tissues, bacteria, and foreign bodies from traumatic tissue by phagocytosis is the most essential defense mechanism of the inflammatory stage [13,14]. New tissue formation is prevented in case of failure in any of these phases. Similar to tissue healing, anastomosis line healing also consists of the same stages, so all systemic and local factors must work correctly. Disruption in these processes may cause increased morbidity and mortality [15]. In this study, we suggest that the effect of NAC on anastomotic healing and HUN could be due to deterioration in these stages of wound healing.

Anastomotic strictures, which occur in the early stages after surgery, often occur due to benign reasons, especially due to the technique applied or the development of ischemia in the tissue [16]. The effect of the anastomosis technique could not be evaluated in this retrospective study because all ureteroileal anastomoses were hand-stitched using the same technique. Ischemic strictures may be due to local and systemic factors such as vascular pedicle injury (arterial supply or venous drainage), excessive tension, extrinsic compression (e.g., cancer recurrence), cardiovascular disease, or perioperative hypotension [16]. Several of these factors may play a role in anastomotic stricture [17]. Consequently, meticulous surgical techniques and the prevention of postoperative hypotension are crucial to minimizing anastomotic stricture's risk.

Collagen formation and storage are essential for completing the healing process [13]. It is theoretically assumed that chemotherapy drugs reduce the proliferation and functioning of fibroblasts by affecting their activity [18-20]. Pramateftakis et al. [21] showed that NAC delays wound healing of intestinal anastomosis by reducing collagen formation in rats and appears dose-related. Ozel et al. [22] demonstrated that the preoperative intravenous administration of 5-fluorouracil chemotherapy negatively affects anastomotic healing. Inui et al. [23] reported that healing of the bronchial anastomosis was closely related to regional mucosal blood flow, and Yamamoto et al. [24] found that preoperative chemotherapy had adversely little effect on bronchial mucosal blood flow and healing of the bronchial stump. In line with these studies, this study demonstrated that NAC may cause deterioration of the ureteroileal anastomosis and HUN.

This study had several limitations. The retrospective design and heterogeneous study population were the main limitations of this study. The low number of cases and the relatively short-term follow-up of the patients are other limitations. Therefore, prospective studies with larger populations are needed. Moreover, the low number of patients who were administered neoadjuvant chemotherapy was another limitation. Although the pT stage was similar between the two groups, the metastatic disease that developed during follow-up was different. However, this study is valuable in investigating the associated factors of postoperative HUN in patients with RC.

## Conclusions

NAC is considered an independent factor in increasing postoperative HUN and the need for revision surgery in patients with BC because of impaired anastomotic healing. Moreover, postoperative metastatic disease is significantly correlated with postoperative HUN; however, multivariable analysis showed that its significance is not as obvious as with NAC.
